# Alcohol consumption and risk of cardiovascular disease, cancer and mortality: a prospective cohort study

**DOI:** 10.1186/s12937-021-00671-y

**Published:** 2021-02-01

**Authors:** Xinyuan Zhang, Yan Liu, Shanshan Li, Alice H. Lichtenstein, Shuohua Chen, Muzi Na, Susan Veldheer, Aijun Xing, Yanxiu Wang, Shouling Wu, Xiang Gao

**Affiliations:** 1 Department of Nutritional Sciences, The Pennsylvania State University, 109 Chandlee Lab, University Park, PA 16802 USA; 2 Department of Cardiology, Kailuan General Hospital, 57 Xinhua East Rd, Tangshan, 063000 China; 3 Slone Epidemiology Center, Boston University School of Medicine, Boston, USA; 4 Jean Mayer USDA Human Nutrition Research Center on Aging, Tufts University, Boston, USA; 5 Department of Family and Community Medicine and Public Health Sciences, The Pennsylvania State University College of Medicine, Hershey, USA

**Keywords:** Alcohol consumption, Cardiovascular disease, Cancer, Mortality, Nutritional epidemiology

## Abstract

**Background:**

Studies regarding whether light to moderate alcohol consumption is associated with a lower risk of cardiovascular diseases (CVD) have generated mixed results. Further, few studies have examined the potential impact of alcohol consumption on diverse disease outcomes simultaneously. We aimed to prospectively study the dose-response association between alcohol consumption and risk of CVD, cancer, and mortality.

**Methods:**

This study included 83,732 adult Chinese participants, free of CVD and cancer at baseline. Participants were categorized into 6 groups based on self-report alcohol consumption: 0, 1–25, 26–150, 151–350, 351–750, and > 750 g alcohol/wk. Incident cases of CVD, cancers, and mortality were confirmed by medical records. Hazard ratios (HRs) for the composite risk of these three outcomes, and each individual outcome, were calculated using Cox proportional hazard model.

**Results:**

During a median follow-up of 10.0 years, there were 6411 incident cases of CVD, 2947 cancers and 6646 deaths. We observed a J-shaped relation between alcohol intake and risk of CVD, cancer, and mortality, with the lowest risk at 25 g/wk., which is equivalent to ~ 2 servings/wk. Compared to consuming 1–25 g/wk., the adjusted HR for composite outcomes was 1.38 (95% confidence interval (CI):1.29–1.49) for non-drinker, 1.15 (95% CI: 1.04–1.27) for 26–150 g/wk., 1.22 (95% CI: 1.10–1.34) for 151–350 g/wk., 1.33 (95% CI: 1.21–1.46) for 351–750 g/wk., and 1.57 (95% CI: 1.30–1.90) for > 750 g/wk., after adjusting for age, sex, lifestyle, social economic status, and medication use.

**Conclusions:**

Light alcohol consumption at ~ 25 g/wk was associated with lower risk of CVD, cancer, and mortality than none or higher consumption in Chinese adults.

**Supplementary Information:**

The online version contains supplementary material available at 10.1186/s12937-021-00671-y.

## Introduction

Worldwide in 2016, current drinkers aged 15 years and over consumed an average of 230 g of alcohol per week [1]. Although the prevalence of alcohol intake worldwide has decreased since 2000, in contrast, China has experienced a decrease in the prevalence of lifetime abstainers and an increase in total alcohol per capita consumption [1].

The association between alcohol consumption and chronic health outcomes is controversial. Early studies have consistently reported that low to moderate alcohol consumption (i.e., < ~ 200 g/wk) is associated with lower risk of cardiovascular disease (CVD) than those who consume no alcohol and or have very high consumption [2–4]. Recently, however, cumulative evidence from combined perspective studies [5] and genetic epidemiological studies [6, 7] have challenged this traditional J-shape relationship, reporting a linear positive association between alcohol intake and CVD risk.

Heavy intake of alcohol has been causally linked to cancers of the oral cavity, pharynx, esophagus, liver, larynx, colorectum, female breast, and possibly to cancers of the pancreas and prostate [8]. In contrast, some observational studies have suggested that light to moderate alcohol is associated with lower risk for total cancer and cancers of lung, cervix, thyroid, and kidney [9].

Although many studies have reported a positive association between alcohol intake and fatalities from accidents, intoxication, indulgence or self-harm, and altered motor function [10], some studies have reported that consumption of up to 100 g/wk. of alcohol was inversely associated with all-cause mortality [11]. Currently there are few available reports on the relationship between alcohol intake and site-specific cancer mortality, with little evidence of a beneficial effect related to low to moderate alcohol intake [11]. This gap makes it difficult to draw conclusions on the relationship between alcohol intake and overall health outcome.

Alcohol consumption, and its impact on chronic diseases and mortality, is heavily influenced by genes, lifestyle, and socio-economic status (SES) [2]. Aldehyde dehydrogenase (ALDH2) deficiency, the lack of a major enzyme responsible for in vivo alcohol oxidation, is common among Asian populations, while being rare in Western populations [12]. Meanwhile, Asian countries rank high in several alcohol-attributed diseases, included cancers of liver and esophagus [13]. The high level of disease risk and genetic predisposition suggests that specific attention to this population is warranted. To date, most cohort studies have been conducted in Western and high-income countries [2–5, 11, 14, 15], leaving Asian and middle- to low-income countries underrepresented. Further, previous meta-analyses and pooled-analyses included populations with vast variability in SES [5, 9, 16–18]. Inclusion of results from a variety of populations and study designs inevitably introduces heterogeneity to the results.

We aim to assess the relationship between alcohol consumption and adverse health outcomes, including cardiovascular diseases, cancers (total and site-specific), and mortality, in a homogenous population. We used a composite of CVD, cancer and total mortality as primary outcomes, to illustrate an overall pattern of alcohol consumption and chronic disease risk, as did previously [19, 20]. As a secondary analysis, associations between alcohol intake and each condition were examined. Our hypothesis was that low to moderate alcohol consumption would be associated with lower risk of major chronic diseases and mortality, compared to none or high alcohol consumption.

## Methods

### Study population

The Kailuan Study is an ongoing prospective cohort based on the Kailuan community in the city of Tangshan, northeast China (trial registration number: ChiCTR-TNRC-11001489) [21]. In brief, 101,510 participants (81,110 men and 20,400 women, 18 years or older) were recruited into the study from June 2006 to October 2007 and were followed-up biennially. At baseline and every follow-up, participants completed a clinical examination, including physical and laboratory measurements, and self-administrated questionnaires. In the current study, we included participants from the 2006–2007 baseline and their follow-up information until 2016–2017.

Exclusion criteria were 1) not answering questions regarding alcohol consumption, or 2) having history of CVD or cancer at baseline. Out of the 101,510 participants, 14,316 had missing response to questions regarding alcohol consumption at baseline (2006–2007). A baseline comparison between these 14,316 excluded participants and included participants was presented in Supplemental Table 1. We further excluded those who had had cardiovascular disease or cancer (*n* = 3845) prior to baseline. Included in the analyses were 83,732 participants (Supplemental Figure 1).

### Assessment of alcohol consumption

A self-administered questionnaire were used to collect information on alcohol consumption [22]. Questions included 1) whether they consumed alcoholic beverages in the past 12 months, 2) if so, the beverage types (beer, wine or hard liquor), and 3) the amount and frequency of intake for each beverage type. Gram (g) of alcohol consumed per week was calculated by multiplying the average frequency (times per week) by the amount usually consumed for each beverage and its average alcohol content (assumed to be 5.0 g for 100 g beer, 12.0 g for 100 g wine, and 40.0 g for 100 g hard liquor). Gram of alcohol was categorized into 5 groups: 1–25, 26–150, 151–350, 351–750, and > 750 g/wk., based on previous published cut-off points [5]. Not drinking any alcoholic beverages in the past 12 months were categorized as 0 g/wk.

Our previous analysis showed a dose-response relationship between alcohol consumption and high-density lipoprotein cholesterol (HDL-C) concentrations in the Kailuan Study participants [22], which further support the validity of the self-reported alcohol consumption data collected as part of the Kailuan study.

### Assessment of outcomes

The primary end point was a composite of CVD, cancer, and mortality, whichever happened first [19, 20]. Secondary outcomes were individual diseases/mortality, including myocardial infarction, stroke, heart failure and atrial fibrillation; alcohol-related cancer (including oral cavity and pharynx, larynx, esophageal, colorectum, anal, salivary gland, liver, and female breast), and other cancers (referred to as “non-alcohol-related cancers”) [9, 23]; and CVD-attributed, cancer-attributed and other-cause mortality.

Incident cases of CVD, cancer, and death were confirmed by review of insurance and medical records, as detailed elsewhere [21, 24, 25]. Questions related to history of CVD and cancer were included in the biennial questionnaire. All participants were linked to the Municipal Social Insurance Institution and the Hospital Discharge Register for incidence of CVD and cancer, which covers all the Kailuan study participants. Medical records from 2006 to 2016 of all patients from the 11 registered hospitals of Kailuan Study were reviewed by study physicians, including 3 cardiologists for CVD ascertainment and 3 oncologists for cancer ascertainment. Heart failure was diagnosed according to Framingham criteria [26]. Atrial fibrillation was diagnosed using a standard 12-lead electrocardiogram [27]. Cancer was diagnosed using pathological and imaging diagnosis and coded by International Classification of Diseases, 10th version (ICD-10) [28]. Mortality information was collected from provincial vital statistics offices. Blinded clinicians reviewed death certificates and coded cause of death according to ICD-10 codes [29].

### Assessment of covariates

We used questionnaires data for information on SES (eg, marital status, education level, occupation type, and household income per capita), medical history, use of medications (eg, anti-hypertensive, anti-diabetes and lipid-lowering agents), and lifestyle factors (eg, smoking status, physical exercise, and salt intake), as detailed elsewhere [21, 28, 29]. Marital status was categorized into single, first marriage, and other; education level was categorized into elementary school or below, middle or high school, or college or above; household income per capita was categorized into ≤500, 501–1000, or > 1000 Chinese yuan/month; smoking status was categorized into never, past, and current smoker; physical activity was categorized into < 1, 1–3, or ≥ 4 times/week; sodium intake was categorized into < 6, 6–9.9, or ≥ 10 g/day.

Clinical examinations were performed at 7–9 am by trained nurses. Anthropometry examinations (eg, height and weight) were conducted. Body mass index (BMI) was calculated as weight (kg)/height^2^ (m^2^). Systolic and diastolic blood pressure were measured on standard desktop mercury sphygmomanometers. Overnight fasting (> 8 h) blood samples were collected to measure serum concentrations of fasting blood glucose (FBG) with the hexokinase/glucose-6-phosphate dehydrogenase method, HDL-C and low- density lipoprotein cholesterol (LDL-C) with direct test assay (Mind Bioengineering Co. Ltd., Shanghai, China), uric acid with commercial kit (Ke Hua Biological Engineering Corporation, Shanghai, China), and high sensitive C-reactive protein (hs-CRP) with high sensitivity nephelometry assay (Cias Latex CRP-H; Kanto Chemical, Tokyo, Japan), which were analyzed by auto-analyzer (Hitachi 747; Hitachi, Tokyo, Japan) at the central laboratory of Kailuan hospital [24, 30]. Concentration of hs-CRP was log-transformed for statistical analysis due to skewed distribution.

### Statistical analysis

Baseline characteristics were compared using one-way Analysis of Variance for continuous variables and chi-square tests for categorical variables.

We computed the person-year of follow-up for each participant that contributed to each outcome from 2006 baseline to the date of diagnosis or the end of follow-up (December 31, 2016), whichever came first.

Hazard ratios (HR) and 95% confidence intervals (CI) for each alcohol consumption category were calculated using a Cox proportional-hazards model, adjusting for age, sex, SES, lifestyle factors, medication history, BMI and FBG concentration. The group 1–25 g/wk. of alcohol consumption was used as reference group. Trends across groups were assessed in models using the median alcohol consumption in each group as a continuous variable. To reduce the possibility of reverse-causality, we further conducted a 2-year lag analysis by excluding incident cases that occurred within the first 2 years of follow-up. The multiplicative interactions were tested between alcohol consumption and age, sex, smoking status, and occupation. Because very low alcohol intake is unlikely to have a biological effect, and the apparent benefit in this group could be due to confounding, we also conducted a sensitivity analysis by excluding participants with 1–4 g alcohol/wk. (i.e., 1 drink/mo).

We further constructed a restricted cubic spline model to display the dose-response association with 95% CIs between continuous alcohol consumption and major chronic disease. Data were analyzed using SAS version 9.4 (SAS Institute Inc., US).

## Results

Of the total 83,732 participants who were included in the analyses, the mean age was 51.3 ± 12.3 years, and 65,081 (77.7%) were men. Baseline characteristics across the alcohol consumption groups are shown in Table 1.

**Table 1:** Baseline characteristic comparison of 83,732 participants according to alcohol consumption

Alcohol consumption, g/wk	0	1–25	26–150	151–350	351–750	> 750
	*n* = 58,706	*n* = 9258	*n* = 5024	*n* = 4520	*n* = 5522	*n* = 702
Age, y	52.12 ± 12.3	46.4 ± 13.3	48.5 ± 12.3	54.5 ± 12.3	50.4 ± 9.1	50.5 ± 8.7
Men, %	69.3	94.4	98.6	99.7	99.9	100.0
Socio-economic status						
Marriage, %						
Single	1.2	5.3	1.9	0.3	0.3	0.3
First marriage	95.2	90.9	93.8	95.2	95.1	94.7
Other	3.6	3.8	4.3	4.5	4.6	5.0
Education, %						
Elementary school or below	9.2	8.9	11.7	18.5	14.3	17.2
Middle or high school	86.0	73.5	73.2	78.3	82.1	79.1
College or above	4.8	17.6	15.1	3.2	3.6	3.7
Occupation type, %						
Coal miner	20.4	48.7	48.3	53.6	59.2	53.2
Blue-collar worker	73.9	40.3	41.0	41.9	37.2	43.4
White-collar worker	5.7	11.0	10.7	4.5	3.6	3.4
Household income per capita, %						
< 500 CNY/month	21.8	40.1	37.1	43.3	45.7	47.7
501–1000 CNY /month	73.5	47.9	50.0	50.2	46.6	42.3
> 1000 CNY /month	4.7	12.0	12.9	6.5	7.7	10.0
Lifestyle						
Beverage types, %						
Wine	–	0.4	0.07	0.01	0.00	0.00
Beer	–	44.2	17.4	1.3	0.7	1.0
Hard liquor	–	53.3	82.2	99.8	100.0	100.0
Smoking status, %						
Never	82.0	30.9	20.4	16.6	11.1	10.0
Past	3.7	9.6	8.9	7.0	5.5	4.7
Current	14.3	59.5	70.7	76.4	83.4	85.3
Physical exercise, %						
< 1 times/week	5.2	14.0	15.9	14.9	19.8	22.7
1–3 times/week	82.5	67.0	65.0	56.2	62.7	51.8
> 4 times/week	12.3	19.0	19.1	28.9	17.5	25.5
Sodium intake, %						
< 6 g/day	7.0	13.9	12.9	13.0	12.5	10.1
6–9.9 g/day	86.3	70.0	69.1	66.3	65.1	57.4
> 10 /day	6.7	16.1	18.0	20.7	22.4	32.5
Medication						
Anti-hypertension drugs	9.3	11.2	13.1	13.8	13.0	16.5
Anti-diabetes drugs	2.4	2.2	2.3	1.9	1.4	1.4
Lipid-lowering drugs	0.7	1.3	1.3	0.7	0.8	0.9
Clinical measurements						
Body mass index, kg/m^2^	25.0 ± 3.6	25.0 ± 3.4	25.5 ± 3.5	24.8 ± 3.2	25.1 ± 3.2	25.2 ± 3.3
Systolic blood pressure, mmHg	131 ± 21	126 ± 19	129 ± 20	136 ± 21	135 ± 20	134 ± 21
Diastolic blood pressure, mmHg	83 ± 12	82 ± 11	83 ± 12	86 ± 12	87 ± 12	86 ± 12
Fasting blood glucose, mmol/L	5.48 ± 1.75	5.39 ± 1.47	5.52 ± 1.63	5.50 ± 1.48	5.56 ± 1.52	5.61 ± 1.57
HDL-C, mmol/L	1.56 ± 0.40	1.47 ± 0.37	1.51 ± 0.38	1.59 ± 0.41	1.62 ± 0.41	1.58 ± 0.40
LDL-C, mmol/L	2.28 ± 0.89	2.48 ± 0.79	2.52 ± 0.86	2.51 ± 0.90	2.56 ± 0.90	2.59 ± 0.91
Uric acid, mmol/L	275 ± 79	307 ± 82	318 ± 86	316 ± 87	318 ± 88	324 ± 86
hs-CRP, mg/L	0.80 (0.30, 2.20)	0.71 (0.30, 1.72)	0.80 (0.30, 1.90)	0.79 (0.31, 1.90)	0.72 (0.30, 1.80)	0.89 (0.39, 2.01)

Values are mean ± standard deviation, median with interquartile range, or frequency (percent)

*CNY* Chinese yuan, *SBP* systolic blood pressure, *DBP* diastolic blood pressure, *FBG* fasting blood glucose, *HDL-C* high-density lipoprotein cholesterol, *LDL-C* low-density lipoprotein cholesterol, *hs-CRP* high-sensitivity C-reactive protein

### Alcohol and CVD, cancer, and mortality

**Fig. 1:**
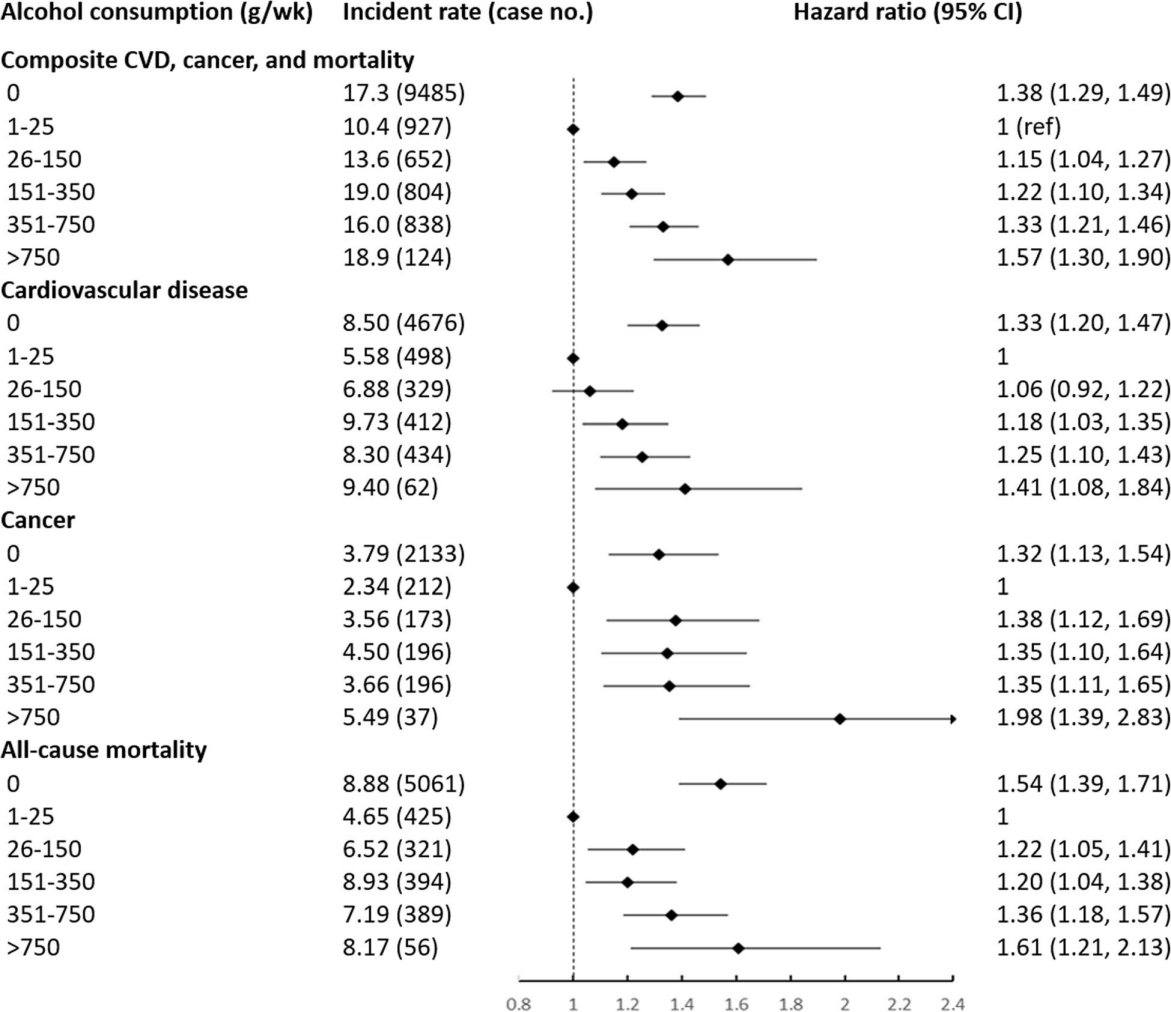
Associations between alcohol consumption and cardiovascular disease, cancer, and all-cause mortality among 83,732 participants, adjusted for age, sex, marriage (single, first marriage, other), education (elementary school or below, middle or high school, college or above), household income per capita (≤500, 501–1000, > 1000 Chinese yuan/month), smoking status (never, past, current),moderate/vigorous physical activity (< 1, 1–3, ≥4 times/week), sodium intake (< 6, 6–9.9, ≥10 g/day), body mass index (kg/m^2^), fasting blood glucose (mmol/L), anti-hypertension drugs (yes, no), anti-diabetes drugs (yes, no), and lipid-lowering drugs (yes, no). The reference category is zero consumption. Diamonds represent hazard ratios (referenced to 1). Horizontal lines represent 95% confidence intervals. Incidence rate is calculated as per 1000 person-years. CVD, cardiovascular diseases

During a mean 10.0 year of follow-up period (interquartile range 9.7 to 10.2 years), there were 6411 CVD cases, 2947 cancer cases and 6646 deaths. Incident case of developing any composite major chronic diseases (CVD and cancer) or mortality was 9485 events. Compared to consuming 1–25 g alcohol/wk., the adjusted HR was 1.15 (95% CI: 1.04–1.27) for 26–150 g/wk., 1.22 (95% CI: 1.10–1.34) for 151–350 g/wk., 1.33 (95% CI: 1.21–1.46) for 351–750 g/wk., and 1.57 (95% CI: 1.30–1.90) for > 750 g/wk. Non-drinkers were associated with higher composite CVD, cancer, and mortality risk (adjusted HR = 1.38; 95% CI: 1.29–1.49, Fig. 1). Consistently, the restricted cubic spline model resulted in a J-shape association with the lowest risk at ~ 25 g/wk. of alcohol intake (p for nonlinearity < 0.001, Supplemental Figure 2a).

### Alcohol and CVD

**Fig. 2:**
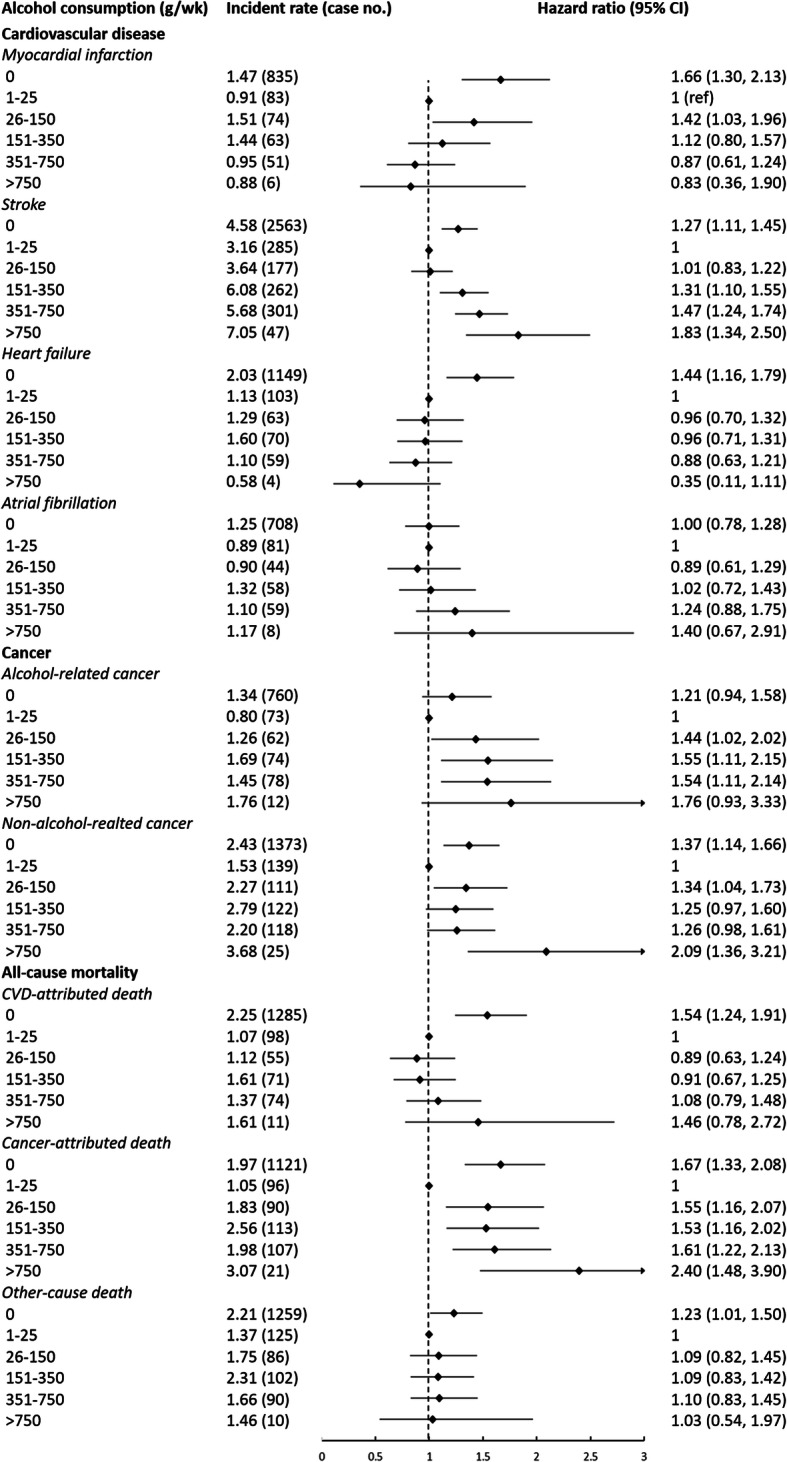
Associations between alcohol consumption and specific chronic diseases and cause-specific mortality among 83,732 participants, adjusted for age, sex, marriage (single, first marriage, other), education (elementary school or below, middle or high school, college or above), household income per capita (≤500, 501–1000, > 1000 Chinese yuan/month), smoking status (never, past, current), moderate/vigorous physical activity (< 1, 1–3, ≥4 times/week), sodium intake (< 6, 6–9.9, ≥10 g/day), body mass index (kg/m^2^), fasting blood glucose (mmol/L), anti-hypertension drugs (yes, no), anti-diabetes drugs (yes, no), and lipid-lowering drugs (yes, no). The reference category is zero consumption. Diamonds represent hazard ratios (referenced to 1). Horizontal lines represent 95% confidence intervals. Incidence rate is calculated as per 1000 person-years. CVD, cardiovascular diseases

Light to moderate alcohol consumption (1–150 g/wk), but not heavy consumption (> 150 g/wk), was significantly associated with lower risk of CVD, relative to non-drinkers (Fig. 1). Alcohol consumption was inversely associated with myocardial infarction and heart failure in a dose-response manner (P_trend_ < 0.001, Fig. 2). In contrast, there was a J-shape association between alcohol intake and stroke risk -- consumption of alcohol 1–150 g/wk. was significantly associated with lower stroke risk and > 350 g/wk. was associated with higher risk (Fig. 2). No significant association between alcohol intake and atrial fibrillation risk was observed (Fig. 2).

### Alcohol and cancer

Individuals consuming 1–25 g alcohol/wk. had the lowest risk compared to other groups (*p* < 0.05 for all, Fig. 1). Alcohol drinkers had marginally significant to significant higher risk of alcohol-attributed site-specific cancers (oral cavity, pharynx, larynx, esophageal, colorectum, anal, salivary gland, liver, and female breast) compared to non-drinkers (P_trend_ = 0.009, Fig. 2). In contrast, risk of non-alcohol-related cancers was significantly higher in non-drinkers, compared to individuals consuming 1–25 g/wk. alcohol (HR = 1.37; 95% CI, 1.14–1.66, Fig. 2).

### Alcohol and mortality

Non-drinkers and alcohol consumption > 25 g/wk. was associated with higher risk of all-cause mortality compared to 1–25 g/wk. (Fig. 1). Restricted cubic spline modeling resulted in a J-shape association (Supplemental Figure 2d). Similar patterns were observed for cause-specific mortality (Fig. 2).

### Secondary analyses

We found a significant interaction between alcohol and smoking (p for interaction < 0.001, Table 2), but not age, sex, and occupation, in relation to the composite CVD, cancer, and mortality risk. Due to small number of participants in > 750 g/wk. category, we combined this category with 351–750 g/wk. category in subgroup analyses. The association between alcohol intake and CVD, cancer, and mortality was stronger for never smokers and past smokers than current smokers (Table 2). For lung cancer specifically, individuals consuming 1–25 g alcohol/wk. had the lowest risk (*p* < 0.05 compared to other groups).

**Table 2:** Association of alcohol consumption and CVD, cancer and mortality in different smoking status

Alcohol consumption, g/wk	0	1–25	26–150	151–350	> 350	P_trend_	P_interaction_
	*n* = 50,291/8379	*n* = 3748/5501	*n* = 1470/3549	*n* = 1066/3449	*n* = 1014/5200		
Composite CVD, cancer, and mortality							< 0.001
Non-smoker	1.43 (1.28, 1.59)	1 (ref)	1.15 (0.97, 1.36)	1.16 (0.97, 1.39)	1.19 (0.98, 1.45)	0.03	
Smoker	1.31 (1.18, 1.44)	1	1.14 (1.01, 1.29)	1.22 (1.09, 1.37)	1.37 (1.23, 1.53)	0.002	
CVD							< 0.001
Non-smoker	1.39 (1.20, 1.62)	1	1.04 (0.81, 1.33)	1.17 (0.91, 1.50)	1.24 (0.94, 1.62)	0.20	
Smoker	1.22 (1.07, 1.40)	1	1.05 (0.89, 1.25)	1.16 (0.99, 1.36)	1.26 (1.08, 1.46)	0.09	
Cancer							0.05
Non-smoker	1.27 (1.01, 1.59)	1	1.59 (1.14, 2.23)	1.21 (0.83, 1.77)	1.03 (0.66, 1.61)	0.005	
Smoker	1.27 (1.03, 1.56)	1	1.28 (0.99, 1.66)	1.33 (1.05, 1.69)	1.48 (1.18, 1.86)	0.005	
Mortality							< 0.001
Non-smoker	1.55 (1.33, 1.81)	1	1.23 (0.97, 1.56)	1.15 (0.90, 1.47)	1.09 (0.81, 1.46)	< 0.001	
Smoker	1.50 (1.30, 1.74)	1	1.21 (1.01, 1.46)	1.22 (1.03, 1.46)	1.45 (1.23, 1.71)	0.35	

Values are hazard ratio and 95% confidence intervals. Numbers of non-smoker/smoker were listed for each category

Adjusted for age, sex, marriage (single, first marriage, other), education (elementary or below, middle or high school, college or above), household income per capita (≤500, 501–1000, > 1000 Chinese yuan/month), moderate/vigorous physical activity (< 1, 1–3, ≥4 times/week), sodium intake (< 6, 6–9.9, ≥10 g/day), body mass index (kg/m^2^), fasting blood glucose (mmol/L), anti-hypertension drugs (yes, no), anti-diabetes drugs (yes, no), and lipid-lowering drugs (yes, no)

*CVD* cardiovascular diseases

The 2-year lag analyses and analyses after excluding former drinkers or very light drinkers (1–4 g/wk) did not significantly change the result (Supplemental Table 2–4). Results of other interaction tests (age, sex, and occupation) and subgroup analyses are presented in Supplemental Tables 5–8. Analyses of different alcoholic beverage types and by cohort-specific tertiles of alcohol consumption are presented in Supplemental Tables 9 & 10.

## Discussion

In this large-scale prospective study including approximately 100,000 Chinese adults, we found that participants who reported consuming 1–150 g/wk. of alcohol had lower risk for CVD, cancer, and mortality, relative to non-drinkers and heavy drinkers. This amount of alcohol intake is equivalent to approximately no more than 10 servings of alcohol/wk. The range identified is consistent with most global dietary guidelines for low-risk drinking cutoffs (100 to 300 g/wk) [31]. However, in this Chinese cohort the lowest risk was observed among those with ~ 25 g/wk., equivalent to ~ 2 servings per week. Because the mechanism for each CVD disorder or site-specific cancer is different, both individual and composite outcomes should be taken into consideration when developing dietary policy. Currently in China, there is no national policy on alcohol sales restrictions and no dietary guidelines for alcohol containing beverages. This study provides evidence for recommending light consumption of alcohol.

Our study in an Asian cohort supports prior observations of a J-shaped association between alcohol consumption and CVD [3, 17]. We found an overall lower CVD risk at consuming 1–150 g alcohol/wk. In secondary analyses, we identified different patterns for specific CVD. A J-shaped curve was found for stroke and an almost linear inverse association was found for myocardial infarction and heart failure. As previously suggested, alcohol could impact CVD risk via its favorable effects of raising HDL-C concentration and reducing inflammation, and its unfavorable effect of increasing blood pressure [6]. However, the impact of these risk factors on different CVDs (i.e., myocardial infarction vs stroke) could be different, thus potentially mediating their association with alcohol consumption. For example, the association of HDL-C and myocardial infarction could be stronger than its association with stroke [5, 32, 33]. Further, high blood pressure has consistently been identified as the strongest risk factor for stroke risk [32]. These factors may contribute to the different patterns in the associations between alcohol and myocardial infarction or heart failure, vs stroke.

We found J-shaped association between alcohol consumption and cancer, especially non-alcohol-related cancers. The causal pathway of alcohol is not fully understood for all cancer types. For female breast cancer, for example, alcohol could increase risk by altering levels of estrogen and estrogen receptors [34]. Hence, the inverse association between light alcohol intake (< 25 g/wk) and overall cancer risk was likely driven by the lower risk of non-alcohol-related cancer. However, limited statistical power due to small number of individual non-alcohol-related cancer cases precluded us from addressing this hypothesis.

Our result showed that light to moderate alcohol intake was consistently associated with lower risk of total mortality and CVD- and cancer-specific mortality. The former observation is consistent with the result from a recent meta-analysis of 87 studies which found that compared to non-drinkers or high alcohol intakes, low-volume alcohol consumers (9.1–174 g alcohol/wk) had 14% lower risk of overall mortality [35].

In our Chinese cohort the association between alcohol consumption and major chronic disease risk was modified by smoking status. Because alcohol consumption and smoking are common addictive behaviors and co-vary, smoking could mask the effect of alcohol alone on health status [36–38]. As expected, although the protective association of light-to-moderate alcohol to CVD, cancer, and mortality was significant in both smokers and non-smokers, it was stronger in non-smokers. However, even for lung cancer, light alcohol consumption still showed lowest risk, compared to non-drinkers and heavy drinkers.

The potential biological mechanism of the overall beneficial effect of alcohol consumption is not fully understood, but may include altering cholesterol concentrations, especially HDL-C and triglyceride concentrations [22, 39], improving insulin sensitivity [39], decreasing the inflammatory process during cell signaling, decreasing platelet aggregation and blood clotting [40], and interaction with genetic variation in alcohol dehydrogenase polymorphism [41]. Although recent genetic epidemiological studies suggested that drinkers with ALDH2 deficiency had a more favorable profile for CVD risk factors, independent of alcohol consumption [6, 7], no relation was reported in non-drinkers.

This study was based on a large, community-based prospective Chinese cohort, with 16,044 incident events. Details on both exposures and outcomes were available, which enabled appropriate model adjustment and a relative comprehensive understanding of outcomes. The work is not without limitations. Potential confounders, such as diet quality, were not measured. To address this limitation, we adjusted for salt intake and other nutrition-related factors such as BMI, FBG, and lipid profiles to control of variation caused by residual confounding. In our previous cross-sectional analysis based on Kailuan Study 2014, salt intake was highly correlated with overall diet quality [21]. Alcohol consumption was calculated based on self-report frequency and usual amount of drinking. This could introduce measurement error, which might attenuate or magnify the association. However, drinking habits and amounts are common conversations with family and friends in many areas of China (e.g., Tangshan city) [42]. Therefore, self-reported consumption could be an acceptable measurement of the true intake with low risk of systematic bias, as supported by a significant correlation between self-report alcohol and longitudinal HDL-C concentration in the Kailuan Study. Of note, HDL-C concentration has been widely used for the validation study of self-report alcohol intake [22, 43]. Binge drinking was not measured in this study. Genetic variation in ALDH2, and consequent metabolic response to alcohol (e.g., skin flash) were not available. The participants of the Kailuan Study lived in a traditional Chinese industrial community, which limits its generalization to other populations. However, the homogenous nature of the study population reduced variance in potential confounding and enhanced internal validity. Finally, due to the nature of Kailuan cohort, women were underrepresented (22.3%), and most women (96.7%) did not report consuming any alcoholic beverages, which is consistent with Chinese national nutritional survey and other reports [42]. This limited our capability to report sex-specific results as primary findings. Because women might be more vulnerable to alcohol due to pregnancy and risk of breast cancer than men [44], and data from a large scale prospective studies on Asian women are lacking on this topic.

## Conclusions

Light to moderate alcohol consumption was associated with an overall beneficial outcome for CVD, cancer, and mortality compared to non-drinkers and heavy consumption. Individuals who consumed ~ 25 g alcohol per wk. had the lowest risk of CVD, cancer, and mortality, relative to their peers.

## Supplementary Information

**Additional file 1 **: **Supplemental Table 1**. Baseline comparison between excluded participants due to missing alcohol consumption data and included participants. **Supplemental Table 2**. Incidence rate and hazard ratios (95% confidence intervals) for CVD, cancer, and mortality by alcohol consumption at baseline. **Supplemental Table 3**. Hazard ratios (95% confidence intervals) for CVD, cancer, and mortality by alcohol consumption, excluding former drinkers (*n*= 80,472). **Supplemental Table 4**. Hazard ratios (95% confidence intervals) for CVD, cancer, and mortality by alcohol consumption, excluding participants with 1-4 g alcohol/wk (*n*= 80,320). **Supplemental Table 5**. Sex-specific association between alcohol consumption and CVD, cancer, and mortality. **Supplemental Table 6**. Association between alcohol consumption and CVD, cancer, and mortality subgroups by smoking status. **Supplemental Table 7**. Hazard ratios (95% confidence intervals) for CVD, cancer, and mortality by alcohol consumption at baseline by age groups. **Supplemental Table 8**. Hazard ratios (95% confidence intervals) for CVD, cancer, and mortality by alcohol consumption at baseline by occupations. **Supplemental Table 9**. Hazard ratios (95% confidence intervals) for CVD, cancer, and mortality associated with alcohol consumption at baseline by beverage types. **Supplemental Table 10**. Hazard ratios (95% confidence intervals) for CVD, cancer, and mortality by alcohol consumption tertiles. **Supplemental Figure 1**. Flowchart of the study. **Supplemental Figure 2**. Restricted cubic spline model.

## Data Availability

Data will be made available from the corresponding author upon reasonable request and approval.

## Abbreviations

CI: Confidence interval
CVD: Cardiovascular disease
HDL-C: High-density lipoprotein cholesterol
HR: Hazard ratio
Hs-CRP: High sensitive C-reactive protein
ICD-10: International Classification of Diseases, 10th version
SES: Socio-economic status
