# Supplementary material for: Alcohol consumption and risk of cardiovascular disease, cancer and mortality: a prospective cohort study
Source: Nutr J. 2021 Feb 1;20:13. doi: 10.1186/s12937-021-00671-y (PMC7852289; doi:10.1186/s12937-021-00671-y)
Supplement: Supplementary file 1 — Additional file 1 : Supplemental Table 1. Baseline comparison between excluded participants due to missing alcohol consumption data and included participants. Supplemental Table 2. Incidence rate and hazard ratios (95% confidence intervals) for CVD, cancer, and mortality by alcohol consumption at baseline. Supplemental Table 3. Hazard ratios (95% confidence intervals) for CVD, cancer, and mortality by alcohol consumption, excluding former drinkers (n= 80,472). Supplemental Table 4. Hazard ratios (95% confidence intervals) for CVD, cancer, and mortality by alcohol consumption, excluding participants with 1-4 g alcohol/wk (n= 80,320). Supplemental Table 5. Sex-specific association between alcohol consumption and CVD, cancer, and mortality. Supplemental Table 6. Association between alcohol consumption and CVD, cancer, and mortality subgroups by smoking status. Supplemental Table 7. Hazard ratios (95% confidence intervals) for CVD, cancer, and mortality by alcohol consumption at baseline by age groups. Supplemental Table 8. Hazard ratios (95% confidence intervals) for CVD, cancer, and mortality by alcohol consumption at baseline by occupations. Supplemental Table 9. Hazard ratios (95% confidence intervals) for CVD, cancer, and mortality associated with alcohol consumption at baseline by beverage types. Supplemental Table 10. Hazard ratios (95% confidence intervals) for CVD, cancer, and mortality by alcohol consumption tertiles. Supplemental Figure 1. Flowchart of the study. Supplemental Figure 2. Restricted cubic spline model. [file 12937_2021_671_MOESM1_ESM.docx]

**Supplemental material for “Alcohol Consumption and Risk of Cardiovascular Disease, Cancer and Mortality: A Prospective Cohort Study”**

**Supplemental Table 1. Baseline comparison between excluded participants due to missing alcohol consumption data and included participants.**

**Supplemental Table 2. Incidence rate and hazard ratios (95% confidence intervals) for CVD, cancer, and mortality by alcohol consumption at baseline.**

**Supplemental Table 3. Hazard ratios (95% confidence intervals) for CVD, cancer, and mortality by alcohol consumption, excluding former drinkers (n=** **80,472).**

**Supplemental Table 4. Hazard ratios (95% confidence intervals) for CVD, cancer, and mortality by alcohol consumption, excluding participants with 1-4 g alcohol/wk (n= 80,320).**

**Supplemental Table 5. Sex-specific association between alcohol consumption and CVD, cancer, and mortality.**

**Supplemental Table 6. Association between alcohol consumption and CVD, cancer, and mortality subgroups by smoking status.**

**Supplemental Table 7. Hazard ratios (95% confidence intervals) for CVD, cancer, and mortality by alcohol consumption at baseline by age groups.**

**Supplemental Table 8. Hazard ratios (95% confidence intervals) for CVD, cancer, and mortality by alcohol consumption at baseline by occupations.**

**Supplemental Table 9. Hazard ratios (95% confidence intervals) for CVD, cancer, and mortality associated with alcohol consumption at baseline by beverage types.**

**Supplemental Table 10. Hazard ratios (95% confidence intervals) for CVD, cancer, and mortality by alcohol consumption tertiles.**

**Supplemental Figure 1. Flowchart of the study.**

**Supplemental Figure 2. Restricted cubic spline model.**

**Supplemental Table 1. Baseline comparison between excluded participants due to missing alcohol consumption data and included participants.**

| Alcohol consumption, g/wk | Missing alcohol consumption | Included | p |
| --- | --- | --- | --- |
|  | n=14316 | n=83732 |  |
| Age, y | 52.8±14.2 | 51.3±12.3 | <0.001 |
| Men, % | 91.2 | 77.7 | <0.001 |
| Socio-economic status |  |  |  |
| Marriage, % |  |  | <0.001 |
| Single | 3.2 | 1.5 |  |
| First marriage | 92.6 | 94.7 |  |
| Other | 4.2 | 3.8 |  |
| Education, % |  |  | <0.001 |
| Elementary school or below | 10.8 | 10.2 |  |
| Middle or high school | 79.0 | 83.2 |  |
| College or above | 10.2 | 6.6 |  |
| Occupation type, % |  |  | <0.001 |
| Coal miner | 44.2 | 29.8 |  |
| Blue-collar worker | 48.0 | 63.8 |  |
| White-collar worker | 7.8 | 6.4 |  |
| Household income per capita, % | |  | <0.001 |
| <=500 RMB/month | 36.3 | 27.7 |  |
| 500-1000 RMB/month | 54.4 | 66.0 |  |
| >1000 RMB/month | 9.3 | 6.3 |  |
| Lifestyle |  |  |  |
| Smoking status, % |  |  | <0.001 |
| Never | 33.1 | 63.8 |  |
| Past | 9.3 | 5.0 |  |
| Current | 57.6 | 31.2 |  |
| Physical exercise, % |  |  | <0.001 |
| <1 times/week | 11.0 | 8.4 |  |
| 1-4 times/week | 70.9 | 76.8 |  |
| >=4 times/week | 18.1 | 14.8 |  |
| Sodium intake, % |  |  | <0.001 |
| <6 gram/day | 11.1 | 8.9 |  |
| 6-10 gram/day | 76.4 | 80.7 |  |
| >=10 gram/day | 12.5 | 10.4 |  |
| Medication |  |  | <0.001 |
| Anti-hypertension drugs | 8.0 | 10.3 |  |
| Anti-diabetes drugs | 1.4 | 2.3 |  |
| Lipid-lowering drugs | 0.53 | 0.78 |  |
| Clinical measurements |  |  |  |
| Body mass index, kg/m^2^ | 25.0±3.4 | 25.0±3.5 | 0.01 |
| Systolic blood pressure, mmHg | 130±20 | 131±21 | <0.001 |
| Diastolic blood pressure, mmHg | 82±12 | 84±12 | <0.001 |
| Fasting blood glucose, mmol/L | 5.39±1.55 | 5.48±1.69 | <0.001 |
| HDL-C, mmol/L | 1.53±0.42 | 1.55±0.40 | <0.001 |
| LDL-C, mmol/L | 2.29±1.07 | 2.35±0.89 | <0.001 |
| Uric acid, mmol/L | 304±89 | 287±83 | <0.001 |
| hs-CRP, mg/L | 0.96 (0.31, 3.60) | 0.80 (0.30, 2.08) | <0.001 |

SBP, systolic blood pressure; DBP, diastolic blood pressure; FBG, fasting blood glucose; HDL-C, high-density lipoprotein cholesterol; LDL-C, low-density lipoprotein cholesterol; hs-CRP, high-sensitivity C-reactive protein.

**Supplemental Table 2. Incidence rate and hazard ratios (95% confidence intervals) for CVD, cancer, and mortality by alcohol consumption at baseline.**

|  | | Alcohol consumption (g/wk) | | | | | | P_trend_ |
| --- | --- | --- | --- | --- | --- | --- | --- | --- |
|  |  | 0 | 1–25 | 26–150 | 151–350 | 351–750 | >750 |  |
|  |  | n=58706 | n=9258 | n=5024 | n=4520 | n=5522 | n=702 |  |
| Composite CVD, cancer, and mortality | Case, n | 9485 | 927 | 652 | 804 | 838 | 124 |  |
|  | Incidence rate, per 1000 person-years | 17.3 | 10.4 | 13.6 | 19.0 | 16.0 | 18.9 |  |
|  | Age- and sex-adjusted | 1.36 (1.27, 1.46) | 1 (ref) | 1.19 (1.08, 1.32) | 1.26 (1.14, 1.38) | 1.42 (1.29, 1.56) | 1.69 (1.40, 2.04) | 0.01 |
|  | Multivariate model | 1.38 (1.29, 1.49) | 1 | 1.15 (1.04, 1.27) | 1.22 (1.10, 1.34) | 1.33 (1.21, 1.46) | 1.57 (1.30, 1.90) | 0.14 |
|  | 2-Year lag | 1.39 (1.29, 1.50) | 1 | 1.17 (1.05, 1.31) | 1.25 (1.12, 1.38) | 1.33 (1.20, 1.48) | 1.62 (1.33, 1.99) | 0.09 |
| CVD | Case, n | 4676 | 498 | 329 | 412 | 434 | 62 |  |
|  | Incidence rate, per 1000 person-years | 8.50 | 5.58 | 6.88 | 9.73 | 8.30 | 9.40 |  |
|  | Age- and sex-adjusted | 1.27 (1.16, 1.40) | 1 | 1.12 (0.97, 1.28) | 1.21 (1.07, 1.38) | 1.34 (1.18, 1.53) | 1.54 (1.18, 2.00) | 0.05 |
|  | Multivariate model | 1.33 (1.20, 1.47) | 1 | 1.06 (0.92, 1.22) | 1.18 (1.03, 1.35) | 1.25 (1.10, 1.43) | 1.41 (1.08, 1.84) | <0.001 |
|  | 2-Year lag | 1.33 (1.19, 1.49) | 1 | 1.07 (0.91, 1.25) | 1.22 (1.05, 1.41) | 1.25 (1.08, 1.44) | 1.46 (1.09, 1.95) | 0.43 |
| Myocardial infarction | Case, n | 835 | 83 | 74 | 63 | 51 | 6 |  |
|  | Incidence rate, per 1000 person-years | 1.47 | 0.91 | 1.51 | 1.44 | 0.95 | 0.88 |  |
|  | Age- and sex-adjusted | 1.43 (1.14, 1.8) | 1 | 1.51 (1.1, 2.06) | 1.12 (0.81, 1.55) | 0.93 (0.66, 1.32) | 0.87 (0.38, 1.99) | 0.002 |
|  | Multivariate model | 1.66 (1.30, 2.13) | 1 | 1.42 (1.03, 1.96) | 1.12 (0.80, 1.57) | 0.87 (0.61, 1.24) | 0.83 (0.36, 1.90) | 0.41 |
|  | 2-Year lag | 1.67 (1.28, 2.16) | 1 | 1.29 (0.91, 1.83) | 1.09 (0.76, 1.56) | 0.75 (0.50, 1.11) | 0.61 (0.22, 1.66) | <0.001 |
| Stroke | Case, n | 2563 | 285 | 177 | 262 | 301 | 47 |  |
|  | Incidence rate, per 1000 person-years | 4.58 | 3.16 | 3.64 | 6.08 | 5.68 | 7.05 |  |
|  | Age- and sex-adjusted | 1.27 (1.13, 1.44) | 1 | 1.05 (0.87, 1.27) | 1.38 (1.17, 1.64) | 1.62 (1.38, 1.91) | 2.03 (1.49, 2.76) | <0.001 |
|  | Multivariate model | 1.27 (1.11, 1.45) | 1 | 1.01 (0.83, 1.22) | 1.31 (1.10, 1.55) | 1.47 (1.24, 1.74) | 1.83 (1.34, 2.50) | <0.001 |
|  | 2-Year lag | 1.25 (1.09, 1.45) | 1 | 1.07 (0.88, 1.32) | 1.36 (1.13, 1.64) | 1.50 (1.25, 1.80) | 1.90 (1.36, 2.65) | <0.001 |
| Heart failure | Case, n | 1149 | 103 | 63 | 70 | 59 | 4 |  |
|  | Incidence rate, per 1000 person-years | 2.03 | 1.13 | 1.29 | 1.60 | 1.10 | 0.58 |  |
|  | Age- and sex-adjusted | 1.36 (1.11, 1.67) | 1 | 1.03 (0.75, 1.4) | 0.96 (0.71, 1.29) | 0.9 (0.66, 1.24) | 0.49 (0.18, 1.34) | <0.001 |
|  | Multivariate model | 1.44 (1.16, 1.79) | 1 | 0.96 (0.70, 1.32) | 0.96 (0.71, 1.31) | 0.88 (0.63, 1.21) | 0.35 (0.11, 1.11) | <0.001 |
|  | 2-Year lag | 1.51 (1.20, 1.91) | 1 | 0.96 (0.68, 1.35) | 0.98 (0.70, 1.36) | 0.82 (0.57, 1.17) | 0.41 (0.13, 1.28) | <0.001 |
| Atrial fibrillation | Case, n | 708 | 81 | 44 | 58 | 59 | 8 |  |
|  | Incidence rate, per 1000 person-years | 1.25 | 0.89 | 0.90 | 1.32 | 1.10 | 1.17 |  |
|  | Age- and sex-adjusted | 1.02 (0.81, 1.28) | 1 | 0.9 (0.63, 1.3) | 0.98 (0.7, 1.37) | 1.18 (0.84, 1.65) | 1.3 (0.63, 2.69) | 0.31 |
|  | Multivariate model | 1.00 (0.78, 1.28) | 1 | 0.89 (0.61, 1.29) | 1.02 (0.72, 1.43) | 1.24 (0.88, 1.75) | 1.40 (0.67, 2.91) | 0.11 |
|  | 2-Year lag | 0.89 (0.65, 1.22) | 1 | 0.70 (0.42, 1.16) | 1.02 (0.66, 1.57) | 1.36 (0.89, 2.06) | 1.86 (0.84, 4.12) | 0.006 |
| Cancer | Case, n | 2133 | 212 | 173 | 196 | 196 | 37 |  |
|  | Incidence rate, per 1000 person-years | 3.79 | 2.34 | 3.56 | 4.50 | 3.66 | 5.49 |  |
|  | Age- and sex-adjusted | 1.25 (1.08, 1.44) | 1 | 1.42 (1.16, 1.74) | 1.44 (1.19, 1.76) | 1.44 (1.19, 1.75) | 2.18 (1.53, 3.09) | <0.001 |
|  | Multivariate model | 1.32 (1.13, 1.54) | 1 | 1.38 (1.12, 1.69) | 1.35 (1.10, 1.64) | 1.35 (1.11, 1.65) | 1.98 (1.39, 2.83) | 0.01 |
|  | 2-Year lag | 1.34 (1.13, 1.59) | 1 | 1.41 (1.13, 1.77) | 1.31 (1.04, 1.64) | 1.38 (1.11, 1.73) | 2.07 (1.40, 3.06) | 0.03 |
| Alcohol-related cancer | Case, n | 760 | 73 | 62 | 74 | 78 | 12 |  |
|  | Incidence rate, per 1000 person-years | 1.34 | 0.80 | 1.26 | 1.69 | 1.45 | 1.76 |  |
|  | Age- and sex-adjusted | 1.22 (0.95, 1.56) | 1 | 1.51 (1.07, 2.11) | 1.66 (1.2, 2.29) | 1.7 (1.24, 2.34) | 2.08 (1.13, 3.83) | <0.001 |
|  | Multivariate model | 1.21 (0.94, 1.58) | 1 | 1.44 (1.02, 2.02) | 1.55 (1.11, 2.15) | 1.54 (1.11, 2.14) | 1.76 (0.93, 3.33) | 0.009 |
|  | 2-Year lag | 1.12 (0.84, 1.50) | 1 | 1.45 (1.00, 2.10) | 1.40 (0.97, 2.02) | 1.49 (1.04, 2.13) | 1.49 (0.71, 3.12) | 0.03 |
| Non-alcohol-related cancer | Case, n | 1373 | 139 | 111 | 122 | 118 | 25 |  |
|  | Incidence rate, per 1000 person-years | 2.43 | 1.53 | 2.27 | 2.79 | 2.20 | 3.68 |  |
|  | Age- and sex-adjusted | 1.27 (1.06, 1.51) | 1 | 1.37 (1.07, 1.76) | 1.34 (1.05, 1.71) | 1.31 (1.03, 1.68) | 2.21 (1.45, 3.39) | 0.02 |
|  | Multivariate model | 1.37 (1.14, 1.66) | 1 | 1.34 (1.04, 1.73) | 1.25 (0.97, 1.60) | 1.26 (0.98, 1.61) | 2.09 (1.36, 3.21) | 0.27 |
|  | 2-Year lag | 1.46 (1.18, 1.81) | 1 | 1.39 (1.05, 1.85) | 1.26 (0.95, 1.67) | 1.32 (1.00, 1.75) | 2.41 (1.52, 3.82) | 0.24 |
| All-cause mortality | Case, n | 5061 | 425 | 321 | 394 | 389 | 56 |  |
|  | Incidence rate, per 1000 person-years | 8.88 | 4.65 | 6.52 | 8.93 | 7.19 | 8.17 |  |
|  | Age- and sex-adjusted | 1.49 (1.35, 1.65) | 1 | 1.25 (1.08, 1.45) | 1.26 (1.10, 1.44) | 1.48 (1.29, 1.70) | 1.75 (1.32, 2.31) | 0.85 |
|  | Multivariate model | 1.54 (1.39, 1.71) | 1 | 1.22 (1.05, 1.41) | 1.20 (1.04, 1.38) | 1.36 (1.18, 1.57) | 1.61 (1.21, 2.13) | 0.40 |
|  | 2-Year lag | 1.54 (1.38, 1.72) | 1 | 1.23 (1.06, 1.44) | 1.22 (1.06, 1.41) | 1.37 (1.18, 1.58) | 1.64 (1.23, 2.20) | 0.54 |
| CVD-attributed mortality | Case, n | 1285 | 98 | 55 | 71 | 74 | 11 |  |
|  | Incidence rate, per 1000 person-years | 2.25 | 1.07 | 1.12 | 1.61 | 1.37 | 1.61 |  |
|  | Age- and sex-adjusted | 1.61 (1.31, 1.98) | 1 | 0.93 (0.67, 1.29) | 0.97 (0.72, 1.32) | 1.25 (0.92, 1.69) | 1.54 (0.82, 2.86) | 0.005 |
|  | Multivariate model | 1.54 (1.24, 1.91) | 1 | 0.89 (0.63, 1.24) | 0.91 (0.67, 1.25) | 1.08 (0.79, 1.48) | 1.46 (0.78, 2.72) | 0.02 |
|  | 2-Year lag | 1.53 (1.23, 1.91) | 1 | 0.91 (0.65, 1.28) | 0.91 (0.66, 1.25) | 1.12 (0.81, 1.54) | 1.39 (0.72, 2.68) | 0.02 |
| Cancer-attributed mortality | Case, n | 1121 | 96 | 90 | 113 | 107 | 21 |  |
|  | Incidence rate, per 1000 person-years | 1.97 | 1.05 | 1.83 | 2.56 | 1.98 | 3.07 |  |
|  | Age- and sex-adjusted | 1.56 (1.26, 1.92) | 1 | 1.58 (1.19, 2.11) | 1.67 (1.27, 2.19) | 1.75 (1.33, 2.30) | 2.76 (1.72, 4.42) | 0.003 |
|  | Multivariate model | 1.67 (1.33, 2.08) | 1 | 1.55 (1.16, 2.07) | 1.53 (1.16, 2.02) | 1.61 (1.22, 2.13) | 2.40 (1.48, 3.90) | 0.11 |
|  | 2-Year lag | 1.71 (1.36, 2.17) | 1 | 1.59 (1.17, 2.16) | 1.53 (1.14, 2.05) | 1.64 (1.22, 2.20) | 2.45 (1.47, 4.09) | 0.17 |
| Other-cause mortality | Case, n | 1259 | 125 | 86 | 102 | 90 | 10 |  |
|  | Incidence rate, per 1000 person-years | 2.21 | 1.37 | 1.75 | 2.31 | 1.66 | 1.46 |  |
|  | Age- and sex-adjusted | 1.26 (1.05, 1.51) | 1 | 1.14 (0.87, 1.50) | 1.10 (0.85, 1.44) | 1.16 (0.89, 1.53) | 1.06 (0.56, 2.02) | 0.32 |
|  | Multivariate model | 1.23 (1.01, 1.50) | 1 | 1.09 (0.82, 1.45) | 1.09 (0.83, 1.42) | 1.10 (0.83, 1.45) | 1.03 (0.54, 1.97) | 0.39 |
|  | 2-Year lag | 1.27 (1.03, 1.56) | 1 | 1.12 (0.84, 1.51) | 1.15 (0.87, 1.52) | 1.09 (0.81, 1.46) | 1.03 (0.52, 2.04) | 0.36 |

Multivariate model adjusted for age, sex, marriage (single, first marriage, other), education (elementary or below, middle or high school, college or above), household income per capita (≤500, 501–1000, >1000 Chinese yuan/month), smoking status (never, past, current), moderate/vigorous physical activity (<1, 1–3, ≥4 times/week), sodium intake (<6, 6–9.9, ≥10 g/day), body mass index (kg/m^2^), fasting blood glucose (mmol/L), anti-hypertension drugs (yes, no), anti-diabetes drugs (yes, no), and lipid-lowering drugs (yes, no).

CVD, cardiovascular diseases.

**Supplemental Table 3. Hazard ratios (95% confidence intervals) for CVD, cancer, and mortality by alcohol consumption, excluding former drinkers (n=** **80,472).**

| Alcohol consumption, g/wk | 0 | 1–25 | 26–150 | 151–350 | 351–750 | >750 | P_trend_ |
| --- | --- | --- | --- | --- | --- | --- | --- |
|  | n=55446 | n=9258 | n=5024 | n=4520 | n=5522 | n=702 |  |
| Composite CVD, cancer, and mortality | 1 (ref) | 0.74 (0.69, 0.80) | 0.85 (0.78, 0.93) | 0.90 (0.83, 0.98) | 0.98 (0.91, 1.07) | 1.17 (0.98, 1.41) | 0.008 |
| CVD | 1 | 0.77 (0.69, 0.85) | 0.81 (0.72, 0.92) | 0.90 (0.81, 1.01) | 0.95 (0.85, 1.07) | 1.09 (0.84, 1.41) | 0.21 |
| Myocardial infarction | 1 | 0.58 (0.46, 0.75) | 0.81 (0.62, 1.06) | 0.65 (0.49, 0.86) | 0.50 (0.36, 0.68) | 0.47 (0.21, 1.07) | <0.001 |
| Stroke | 1 | 0.81 (0.71, 0.93) | 0.81 (0.69, 0.95) | 1.04 (0.90, 1.20) | 1.16 (1.01, 1.34) | 1.48 (1.10, 2.00) | <0.001 |
| Heart failure | 1 | 0.70 (0.56, 0.87) | 0.67 (0.51, 0.89) | 0.67 (0.52, 0.87) | 0.60 (0.45, 0.80) | 0.24 (0.08, 0.76) | <0.001 |
| Atrial fibrillation | 1 | 0.99 (0.77, 1.29) | 0.90 (0.64, 1.25) | 1.03 (0.76, 1.39) | 1.26 (0.93, 1.71) | 1.41 (0.69, 2.88) | 0.10 |
| Cancer | 1 | 0.79 (0.67, 0.92) | 1.09 (0.92, 1.29) | 1.06 (0.91, 1.26) | 1.07 (0.90, 1.27) | 1.57 (1.12, 2.21) | 0.003 |
| Alcohol-related | 1 | 0.84 (0.64, 1.10) | 1.20 (0.90, 1.61) | 1.29 (0.98, 1.70) | 1.26 (0.95, 1.67) | 1.45 (0.79, 2.69) | 0.007 |
| Non-alcohol-related | 1 | 0.76 (0.63, 0.93) | 1.03 (0.83, 1.28) | 0.96 (0.78, 1.18) | 0.98 (0.79, 1.21) | 1.63 (1.08, 2.46) | 0.10 |
| Mortality | 1 | 0.68 (0.61, 0.75) | 0.82 (0.73, 0.93) | 0.81 (0.73, 0.91) | 0.92 (0.82, 1.03) | 1.09 (0.83, 1.43) | 0.69 |
| CVD-attributed | 1 | 0.67 (0.53, 0.83) | 0.59 (0.44, 0.78) | 0.60 (0.46, 0.77) | 0.70 (0.54, 0.92) | 0.95 (0.52, 1.75) | 0.05 |
| Cancer-attributed | 1 | 0.66 (0.52, 0.82) | 1.02 (0.81, 1.29) | 1.02 (0.82, 1.27) | 1.08 (0.86, 1.35) | 1.61 (1.02, 2.54) | 0.01 |
| Other-cause | 1 | 0.79 (0.65, 0.97) | 0.86 (0.68, 1.09) | 0.86 (0.69, 1.07) | 0.86 (0.67, 1.09) | 0.81 (0.43, 1.52) | 0.36 |

Multivariate model adjusted for age, sex, marriage (single, first marriage, other), education (elementary or below, middle or high school, college or above), household income per capita (≤500, 501–1000, >1000 Chinese yuan/month), smoking status (never, past, current), moderate/vigorous physical activity (<1, 1–3, ≥4 times/week), sodium intake (<6, 6–9.9, ≥10 g/day), body mass index (kg/m^2^), fasting blood glucose (mmol/L), anti-hypertension drugs (yes, no), anti-diabetes drugs (yes, no), and lipid-lowering drugs (yes, no).

CVD, cardiovascular diseases.

**Supplemental Table 4. Hazard ratios (95% confidence intervals) for CVD, cancer, and mortality by alcohol consumption, excluding participants with 1-4 g alcohol/wk (n= 80,320).**

| Alcohol consumption, g/wk | 0 | 5–25 | 26–150 | 151–350 | 351–750 | >750 | Ptrend |
| --- | --- | --- | --- | --- | --- | --- | --- |
|  | n=58706 | n=5846 | n=5024 | n=4520 | n=5522 | n=702 |  |
| CVD, cancer, and mortality | 1 (ref) | 0.73 (0.68, 0.80) | 0.83 (0.76, 0.90) | 0.88 (0.81, 0.95) | 0.96 (0.89, 1.04) | 1.13 (0.95, 1.36) | 0.04 |
| CVD | 1 | 0.77 (0.69, 0.86) | 0.80 (0.71, 0.90) | 0.89 (0.80, 0.99) | 0.95 (0.85, 1.06) | 1.06 (0.82, 1.38) | 0.09 |
| Myocardial infarction | 1 | 0.60 (0.45, 0.80) | 0.85 (0.66, 1.10) | 0.67 (0.51, 0.88) | 0.52 (0.38, 0.71) | 0.50 (0.22, 1.12) | <0.001 |
| Stroke | 1 | 0.79 (0.68, 0.92) | 0.79 (0.67, 0.93) | 1.03 (0.89, 1.18) | 1.16 (1.01, 1.32) | 1.44 (1.07, 1.94) | 0.03 |
| Heart failure | 1 | 0.73 (0.57, 0.93) | 0.67 (0.51, 0.87) | 0.67 (0.52, 0.86) | 0.61 (0.46, 0.80) | 0.24 (0.08, 0.76) | <0.001 |
| Atrial fibrillation | 1 | 1.05 (0.79, 1.39) | 0.89 (0.64, 1.23) | 1.02 (0.76, 1.36) | 1.24 (0.93, 1.67) | 1.40 (0.69, 2.89) | 0.26 |
| Cancer | 1 | 0.76 (0.64, 0.91) | 1.05 (0.88, 1.24) | 1.02 (0.87, 1.20) | 1.03 (0.87, 1.21) | 1.51 (1.07, 2.11) | 0.22 |
| Alcohol-related | 1 | 0.80 (0.59, 1.09) | 1.18 (0.89, 1.57) | 1.27 (0.98, 1.66) | 1.27 (0.97, 1.66) | 1.45 (0.79, 2.67) | 0.02 |
| Non-alcohol-related | 1 | 0.74 (0.60, 0.92) | 0.98 (0.80, 1.21) | 0.91 (0.74, 1.11) | 0.92 (0.75, 1.13) | 1.53 (1.02, 2.29) | 0.84 |
| Mortality | 1 | 0.67 (0.60, 0.75) | 0.79 (0.70, 0.89) | 0.78 (0.70, 0.87) | 0.88 (0.79, 0.99) | 1.05 (0.80, 1.37) | <0.001 |
| CVD-attributed | 1 | 0.66 (0.52, 0.85) | 0.58 (0.43, 0.77) | 0.59 (0.46, 0.77) | 0.70 (0.54, 0.92) | 0.95 (0.52, 1.73) | <0.001 |
| Cancer-attributed | 1 | 0.64 (0.50, 0.82) | 0.93 (0.74, 1.17) | 0.92 (0.75, 1.14) | 0.97 (0.78, 1.21) | 1.44 (0.92, 2.27) | 0.96 |
| Other-cause | 1 | 0.85 (0.69, 1.07) | 0.89 (0.70, 1.12) | 0.88 (0.71, 1.10) | 0.89 (0.70, 1.13) | 0.84 (0.45, 1.57) | 0.17 |

Values are hazard ratios and 95% confidence intervals.

Multivariate model adjusted for age, sex, marriage (single, first marriage, other), education (elementary or below, middle or high school, college or above), household income per capita (≤500, 501–1000, >1000 Chinese yuan/month), smoking status (never, past, current), moderate/vigorous physical activity (<1, 1–3, ≥4 times/week), sodium intake (<6, 6–9.9, ≥10 g/day), body mass index (kg/m^2^), fasting blood glucose (mmol/L), anti-hypertension drugs (yes, no), anti-diabetes drugs (yes, no), and lipid-lowering drugs (yes, no).

CVD, cardiovascular diseases.

**Supplemental Table 5. Sex-specific association between alcohol consumption and CVD, cancer, and mortality.**

| Alcohol consumption, g/wk | 0 | 1–25 | 26–150 | 151–350 | 351–750 | >750 | P_trend_ |
| --- | --- | --- | --- | --- | --- | --- | --- |
| Men |  |  |  |  |  |  |  |
| CVD, cancer, and mortality | 1 (ref) | 0.72 (0.67, 0.77) | 0.83 (0.76, 0.90) | 0.88 (0.81, 0.95) | 0.96 (0.89, 1.04) | 1.13 (0.95, 1.36) | 0.12 |
| CVD | 1 | 0.74 (0.67, 0.82) | 0.80 (0.71, 0.90) | 0.89 (0.80, 1.00) | 0.94 (0.84, 1.04) | 1.06 (0.82, 1.37) | 0.49 |
| Myocardial infarction | 1 | 0.61 (0.47, 0.78) | 0.86 (0.67, 1.12) | 0.69 (0.52, 0.90) | 0.52 (0.39, 0.71) | 0.50 (0.22, 1.12) | 0.001 |
| Stroke | 1 | 0.77 (0.68, 0.89) | 0.79 (0.68, 0.93) | 1.03 (0.90, 1.18) | 1.15 (1.01, 1.32) | 1.44 (1.07, 1.93) | <0.001 |
| Heart failure | 1 | 0.68 (0.54, 0.84) | 0.65 (0.50, 0.86) | 0.67 (0.52, 0.87) | 0.60 (0.45, 0.80) | 0.24 (0.08, 0.76) | <0.001 |
| Atrial fibrillation | 1 | 0.98 (0.76, 1.26) | 0.86 (0.62, 1.20) | 1.02 (0.76, 1.36) | 1.22 (0.91, 1.64) | 1.39 (0.68, 2.83) | 0.12 |
| Cancer | 1 | 0.74 (0.63, 0.87) | 1.05 (0.88, 1.24) | 1.01 (0.86, 1.19) | 1.02 (0.87, 1.21) | 1.50 (1.07, 2.10) | 0.02 |
| Alcohol-related | 1 | 0.79 (0.60, 1.04) | 1.18 (0.88, 1.57) | 1.25 (0.96, 1.63) | 1.24 (0.94, 1.62) | 1.40 (0.76, 2.58) | 0.01 |
| Non-alcohol-related | 1 | 0.72 (0.59, 0.88) | 0.99 (0.80, 1.22) | 0.91 (0.74, 1.11) | 0.92 (0.75, 1.14) | 1.54 (1.03, 2.32) | 0.24 |
| Mortality | 1 | 0.65 (0.59, 0.73) | 0.79 (0.70, 0.89) | 0.78 (0.70, 0.87) | 0.88 (0.79, 0.99) | 1.04 (0.79, 1.36) | 0.39 |
| CVD-attributed | 1 | 0.66 (0.53, 0.82) | 0.57 (0.42, 0.75) | 0.59 (0.46, 0.76) | 0.70 (0.54, 0.91) | 0.93 (0.51, 1.70) | 0.01 |
| Cancer-attributed | 1 | 0.61 (0.49, 0.76) | 0.92 (0.73, 1.16) | 0.92 (0.75, 1.14) | 0.97 (0.78, 1.21) | 1.45 (0.92, 2.28) | 0.10 |
| Other-cause | 1 | 0.83 (0.68, 1.01) | 0.89 (0.70, 1.12) | 0.88 (0.71, 1.10) | 0.89 (0.70, 1.13) | 0.83 (0.44, 1.57) | 0.37 |
| Women |  |  |  |  |  |  |  |
| CVD, cancer, and mortality | 1 (ref) | 1.07 (0.75, 1.53) | 1.13 (0.53, 2.37) | 0.50 (0.07, 3.59) | - | - | 0.35 |
| CVD | 1 | 1.13 (0.67, 1.90) | 0.30 (0.04, 2.14) | - | - | - | 0.25 |
| Myocardial infarction | 1 | 0.41 (0.06, 2.99) | - | - | - | - | 0.32 |
| Stroke | 1 | 1.21 (0.59, 2.48) | - | - | - | - | 0.31 |
| Heart failure | 1 | 1.31 (0.53, 3.24) | 1.17 (0.16, 8.48) | - | - | - | 0.64 |
| Atrial fibrillation | 1 | 1.31 (0.40, 4.27) | 2.08 (0.28, 15.67) | - | - | - | 0.86 |
| Cancer | 1 | 1.23 (0.74, 2.05) | 1.49 (0.47, 4.69) | - | - | - | 0.57 |
| Alcohol-related | 1 | 1.32 (0.61, 2.85) | 1.22 (0.17, 8.82) | - | - | - | 0.73 |
| Non-alcohol-related | 1 | 1.15 (0.58, 2.28) | 1.67 (0.41, 6.84) | - | - | - | 0.66 |
| Mortality | 1 | 0.39 (0.15, 1.06) | 1.43 (0.53, 3.90) | 1.09 (0.15, 7.97) | - | - | 0.85 |
| CVD-attributed | 1 | - | 1.51 (0.20, 11.42) | - | - | - | 0.64 |
| Cancer-attributed | 1 | 0.28 (0.04, 2.04) | 2.91 (0.70, 12.12) | - | - | - | 0.75 |
| Other-cause | 1 | - | 1.69 (0.23, 12.54) | - | - | - | 0.66 |

Values are hazard ratios and 95% confidence intervals.

Adjusted for age, marriage (single, first marriage, other), education (elementary or below, middle or high school, college or above), household income per capita (≤500, 501–1000, >1000 Chinese yuan/month), smoking status (never, past, current), moderate/vigorous physical activity (<1, 1–3, ≥4 times/week), sodium intake (<6, 6–9.9, ≥10 g/day), body mass index (kg/m^2^), fasting blood glucose (mmol/L), anti-hypertension drugs (yes, no), anti-diabetes drugs (yes, no), and lipid-lowering drugs (yes, no).

CVD, cardiovascular diseases.

**Supplemental Table 6. Association between alcohol consumption and CVD, cancer, and mortality subgroups by smoking status.**

| Alcohol consumption, g/wk | 0 | 1–25 | 26–150 | 151–350 | >350 | P_trend_ | P_interaction_ |
| --- | --- | --- | --- | --- | --- | --- | --- |
| **CVD** |  |  |  |  |  |  | <0.001 |
| Myocardial infarction |  |  |  |  |  |  | 0.03 |
| Non-smoker | 1.80 (1.21, 2.67) | 1 (ref) | 0.77 (0.37, 1.59) | 1.25 (0.65, 2.39) | 0.48 (0.17, 1.38) | 0.004 |  |
| Smoker | 1.51 (1.10, 2.07) | 1 | 1.65 (1.14, 2.38) | 1.07 (0.72, 1.59) | 0.90 (0.61, 1.33) | 0.004 |  |
| Stroke |  |  |  |  |  |  | 0.05 |
| Non-smoker | 1.31 (1.07, 1.60) | 1 | 1.06 (0.76, 1.48) | 1.21 (0.87, 1.68) | 1.48 (1.06, 2.07) | 0.48 |  |
| Smoker | 1.18 (0.99, 1.42) | 1 | 0.97 (0.77, 1.22) | 1.31 (1.07, 1.61) | 1.49 (1.23, 1.80) | <0.001 |  |
| Heart failure |  |  |  |  |  |  | 0.23 |
| Non-smoker | 1.47 (1.08, 2.01) | 1 | 0.85 (0.49, 1.47) | 0.87 (0.49, 1.52) | 0.88 (0.47, 1.68) | 0.01 |  |
| Smoker | 1.37 (1.01, 1.86) | 1 | 1.00 (0.67, 1.49) | 0.99 (0.68, 1.45) | 0.80 (0.54, 1.17) | 0.001 |  |
| Atrial fibrillation |  |  |  |  |  |  | 0.33 |
| Non-smoker | 1.23 (0.86, 1.75) | 1 | 1.09 (0.62, 1.93) | 1.18 (0.67, 2.06) | 1.50 (0.83, 2.73) | 0.49 |  |
| Smoker | 0.76 (0.52, 1.10) | 1 | 0.72 (0.44, 1.19) | 0.87 (0.56, 1.35) | 1.09 (0.72, 1.65) | 0.09 |  |
| **Cancer** |  |  |  |  |  |  | 0.05 |
| Alcohol-related |  |  |  |  |  |  | 0.28 |
| Non-smoker | 1.27 (0.85, 1.89) | 1 | 2.28 (1.33, 3.91) | 1.17 (0.59, 2.32) | 1.04 (0.47, 2.29) | 0.03 |  |
| Smoker | 1.16 (0.81, 1.65) | 1 | 1.11 (0.71, 1.72) | 1.60 (1.08, 2.37) | 1.59 (1.10, 2.30) | 0.006 |  |
| Non-alcohol-related |  |  |  |  |  |  | 0.1 |
| Non-smoker | 1.27 (0.96, 1.68) | 1 | 1.28 (0.83, 1.98) | 1.24 (0.79, 1.95) | 1.03 (0.60, 1.77) | 0.87 |  |
| Smoker | 1.32 (1.02, 1.71) | 1 | 1.38 (1.01, 1.88) | 1.20 (0.88, 1.62) | 1.42 (1.07, 1.89) | 0.14 |  |
| **Mortality** |  |  |  |  |  |  | <0.001 |
| CVD-attributed |  |  |  |  |  |  | <0.001 |
| Non-smoker | 2.01 (1.43, 2.84) | 1 | 0.98 (0.55, 1.74) | 1.00 (0.56, 1.79) | 0.86 (0.42, 1.80) | 0.002 |  |
| Smoker | 1.22 (0.91, 1.64) | 1 | 0.82 (0.54, 1.23) | 0.84 (0.58, 1.23) | 1.07 (0.76, 1.51) | 0.62 |  |
| Cancer-attributed |  |  |  |  |  |  | 0.51 |
| Non-smoker | 1.37 (0.99, 1.90) | 1 | 1.58 (0.99, 2.54) | 1.16 (0.69, 1.95) | 1.51 (0.87, 2.60) | 0.48 |  |
| Smoker | 1.81 (1.33, 2.45) | 1 | 1.59 (1.10, 2.30) | 1.72 (1.22, 2.43) | 1.89 (1.35, 2.63) | 0.07 |  |
| Other-cause |  |  |  |  |  |  | 0.06 |
| Non-smoker | 1.34 (1.00, 1.78) | 1 | 0.97 (0.60, 1.57) | 1.25 (0.80, 1.96) | 0.79 (0.42, 1.47) | 0.12 |  |
| Smoker | 1.11 (0.84, 1.46) | 1 | 1.12 (0.79, 1.59) | 1.01 (0.72, 1.41) | 1.09 (0.79, 1.49) | 0.04 |  |

Values are hazard ratios and 95% confidence intervals.

Adjusted for age, sex, marriage (single, first marriage, other), education (elementary or below, middle or high school, college or above), household income per capita (≤500, 501–1000, >1000 Chinese yuan/month), moderate/vigorous physical activity (<1, 1–3, ≥4 times/week), sodium intake (<6, 6–9.9, ≥10 g/day), body mass index (kg/m^2^), fasting blood glucose (mmol/L), anti-hypertension drugs (yes, no), anti-diabetes drugs (yes, no), and lipid-lowering drugs (yes, no).

CVD, cardiovascular diseases.

**Supplemental Table 7. Hazard ratios (95% confidence intervals) for CVD, cancer, and mortality by alcohol consumption at baseline by age groups.**

| Alcohol consumption, g/wk | 0 | 1–25 | 26–150 | 151–350 | >350 | P_trend_ | P_interaction_ |
| --- | --- | --- | --- | --- | --- | --- | --- |
| Composite CVD, cancer, and mortality |  |  |  |  |  |  | 0.44 |
| Age <50y | 1.35 (1.20, 1.52) | 1 (ref) | 1.23 (1.05, 1.43) | 1.16 (0.98, 1.36) | 1.35 (1.18, 1.54) | 0.09 |  |
| Age ≥50y | 1.38 (1.26, 1.52) | 1 | 1.08 (0.95, 1.24) | 1.22 (1.08, 1.38) | 1.33 (1.17, 1.51) | 0.02 |  |
| CVD |  |  |  |  |  |  | 0.10 |
| Age <50y | 1.23 (1.05, 1.44) | 1 | 1.11 (0.91, 1.37) | 0.97 (0.77, 1.22) | 1.17 (0.97, 1.40) | 0.92 |  |
| Age ≥50y | 1.35 (1.18, 1.54) | 1 | 1.00 (0.83, 1.22) | 1.24 (1.05, 1.47) | 1.27 (1.06, 1.53) | 0.93 |  |
| Cancer |  |  |  |  |  |  | 0.002 |
| Age <50y | 1.25 (1.00, 1.57) | 1 | 1.31 (0.97, 1.76) | 1.35 (0.99, 1.85) | 1.43 (1.09, 1.86) | 0.03 |  |
| Age ≥50y | 1.28 (1.04, 1.58) | 1 | 1.43 (1.08, 1.89) | 1.23 (0.94, 1.59) | 1.30 (0.98, 1.71) | 0.42 |  |
| Mortality |  |  |  |  |  |  | 0.56 |
| Age <50y | 1.66 (1.37, 2.01) | 1 | 1.31 (1.01, 1.68) | 1.22 (0.93, 1.60) | 1.55 (1.24, 1.93) | 0.54 |  |
| Age ≥50y | 1.50 (1.33, 1.71) | 1 | 1.18 (0.98, 1.41) | 1.20 (1.02, 1.41) | 1.31 (1.10, 1.56) | 0.16 |  |

Adjusted for age, sex, marriage (single, first marriage, other), education (elementary or below, middle or high school, college or above), household income per capita (≤500, 501–1000, >1000 Chinese yuan/month), smoking status (never, past, current), moderate/vigorous physical activity (<1, 1–3, ≥4 times/week), sodium intake (<6, 6–9.9, ≥10 g/day), body mass index (kg/m^2^), fasting blood glucose (mmol/L), anti-hypertension drugs (yes, no), anti-diabetes drugs (yes, no), and lipid-lowering drugs (yes, no).

CVD, cardiovascular diseases.

**Supplemental Table 8. Hazard ratios (95% confidence intervals) for CVD, cancer, and mortality by alcohol consumption at baseline by occupations.**

| Alcohol consumption, g/wk | 0 | 1–25 | 26–150 | 151–350 | >350 | P_trend_ | P_interaction_ |
| --- | --- | --- | --- | --- | --- | --- | --- |
| Composite CVD, cancer, and mortality |  |  |  |  |  |  | 0.19 |
| Coal miner | 1.36 (1.22, 1.52) | 1 (ref) | 1.15 (0.99, 1.33) | 1.13 (0.99, 1.31) | 1.35 (1.19, 1.54) | 0.59 |  |
| Other occupation | 1.42 (1.29, 1.57) | 1 | 1.15 (1.00, 1.32) | 1.31 (1.15, 1.49) | 1.38 (1.21, 1.58) | 0.09 |  |
| CVD |  |  |  |  |  |  | 0.36 |
| Coal miner | 1.28 (1.10, 1.48) | 1 | 1.10 (0.90, 1.35) | 1.13 (0.93, 1.37) | 1.20 (1.01, 1.44) | 0.75 |  |
| Other occupation | 1.39 (1.21, 1.59) | 1 | 1.02 (0.84, 1.24) | 1.25 (1.04, 1.50) | 1.36 (1.14, 1.64) | 0.18 |  |
| Cancer |  |  |  |  |  |  | 0.59 |
| Coal miner | 1.22 (0.97, 1.53) | 1 | 1.31 (0.97, 1.77) | 1.18 (0.89, 1.58) | 1.48 (1.14, 1.92) | 0.03 |  |
| Other occupation | 1.40 (1.13, 1.72) | 1 | 1.43 (1.09, 1.89) | 1.50 (1.14, 1.97) | 1.34 (1.02, 1.78) | 0.18 |  |
| Mortality |  |  |  |  |  |  | 0.72 |
| Coal miner | 1.53 (1.31, 1.78) | 1 | 1.21 (0.97, 1.49) | 1.14 (0.93, 1.40) | 1.44 (1.19, 1.74) | 0.76 |  |
| Other occupation | 1.59 (1.38, 1.84) | 1 | 1.24 (1.01, 1.52) | 1.28 (1.05, 1.55) | 1.36 (1.11, 1.66) | 0.39 |  |

Adjusted for age, sex, marriage (single, first marriage, other), education (elementary or below, middle or high school, college or above), household income per capita (≤500, 501–1000, >1000 Chinese yuan/month), smoking status (never, past, current), moderate/vigorous physical activity (<1, 1–3, ≥4 times/week), sodium intake (<6, 6–9.9, ≥10 g/day), body mass index (kg/m^2^), fasting blood glucose (mmol/L), anti-hypertension drugs (yes, no), anti-diabetes drugs (yes, no), and lipid-lowering drugs (yes, no).

CVD, cardiovascular diseases.

**Supplemental Table 9. Hazard ratios (95% confidence intervals) for CVD, cancer, and mortality associated with alcohol consumption at baseline by beverage types.**

| Beer (n=5070) |  |  |  |  |  |  |
| --- | --- | --- | --- | --- | --- | --- |
| Alcohol consumption, g/wk | 0 | 1–25 | 26–150 | 151–350 | >350 | P_trend_ |
|  | n=58706 | n=4092 | n=875 | n=57 | n=46 |  |
| CVD, cancer, and mortality | 1 (ref) | 0.69 (0.61, 0.78) | 0.83 (0.67, 1.01) | 0.61 (0.27, 1.36) | 0.82 (0.34, 1.96) | 0.07 |
| CVD | 1 | 0.76 (0.64, 0.89) | 0.89 (0.68, 1.17) | 0.62 (0.20, 1.93) | 0.34 (0.05, 2.42) | 0.07 |
| Myocardial infarction | 1 | 0.59 (0.39, 0.88) | 0.58 (0.27, 1.22) | 1.05 (0.15, 7.51) | - | 0.25 |
| Stroke | 1 | 0.80 (0.65, 0.99) | 0.91 (0.64, 1.31) | 0.38 (0.05, 2.68) | 0.59 (0.08, 4.21) | 0.20 |
| Heart failure | 1 | 0.67 (0.47, 0.97) | 0.80 (0.43, 1.51) | - | - | 0.11 |
| Atrial fibrillation | 1 | 1.06 (0.71, 1.60) | 1.24 (0.61, 2.51) | 1.77 (0.25, 12.63) | - | 0.92 |
| Cancer | 1 | 0.71 (0.56, 0.91) | 1.15 (0.80, 1.67) | - | 2.26 (0.73, 7.04) | 0.49 |
| Alcohol-related | 1 | 0.77 (0.50, 1.17) | 1.49 (0.83, 2.68) | - | 2.43 (0.34, 17.40) | 0.69 |
| Non-alcohol-related | 1 | 0.69 (0.51, 0.93) | 1.00 (0.62, 1.61) | - | 2.16 (0.54, 8.69) | 0.58 |
| Mortality | 1 | 0.55 (0.46, 0.67) | 0.75 (0.55, 1.02) | 0.66 (0.21, 2.04) | 1.21 (0.39, 3.75) | 0.17 |
| CVD-attributed | 1 | 0.61 (0.42, 0.90) | 0.56 (0.27, 1.19) | - | 1.91 (0.27, 13.65) | 0.37 |
| Cancer-attributed | 1 | 0.41 (0.27, 0.64) | 0.86 (0.49, 1.50) | - | 1.24 (0.17, 8.84) | 0.33 |
| Other-cause | 1 | 0.78 (0.56, 1.10) | 1.00 (0.56, 1.77) | - | 1.94 (0.27, 13.82) | 0.74 |
| Liquor (n=19796) |  |  |  |  |  |  |
| Alcohol consumption, g/wk | 0 | 1–25 | 26–150 | 151–350 | >350 | P for trend |
|  | n=58706 | n=4933 | n=4128 | n=4511 | n=6224 |  |
| CVD, cancer, and mortality | 1 (ref) | 0.74 (0.68, 0.80) | 0.82 (0.75, 0.90) | 0.88 (0.82, 0.95) | 0.98 (0.91, 1.07) | 0.34 |
| CVD | 1 | 0.76 (0.67, 0.85) | 0.78 (0.68, 0.89) | 0.90 (0.81, 1.00) | 0.97 (0.87, 1.07) | 0.63 |
| Myocardial infarction | 1 | 0.64 (0.48, 0.85) | 0.91 (0.69, 1.19) | 0.68 (0.52, 0.89) | 0.52 (0.39, 0.70) | <0.001 |
| Stroke | 1 | 0.78 (0.67, 0.92) | 0.77 (0.64, 0.92) | 1.04 (0.90, 1.19) | 1.20 (1.05, 1.36) | <0.001 |
| Heart failure | 1 | 0.68 (0.52, 0.88) | 0.63 (0.47, 0.85) | 0.67 (0.52, 0.87) | 0.57 (0.43, 0.75) | <0.001 |
| Atrial fibrillation | 1 | 1.00 (0.74, 1.35) | 0.81 (0.56, 1.17) | 1.03 (0.77, 1.38) | 1.27 (0.96, 1.69) | 0.09 |
| Cancer | 1 | 0.76 (0.63, 0.91) | 0.99 (0.83, 1.19) | 1.02 (0.86, 1.20) | 1.07 (0.92, 1.25) | 0.03 |
| Alcohol-related | 1 | 0.81 (0.59, 1.12) | 1.07 (0.78, 1.47) | 1.25 (0.95, 1.63) | 1.25 (0.96, 1.61) | 0.02 |
| Non-alcohol-related | 1 | 0.73 (0.58, 0.92) | 0.95 (0.76, 1.19) | 0.91 (0.74, 1.12) | 0.99 (0.81, 1.20) | 0.35 |
| Mortality | 1 | 0.70 (0.62, 0.79) | 0.79 (0.70, 0.90) | 0.78 (0.70, 0.87) | 0.90 (0.81, 1.00) | 0.13 |
| CVD-attributed | 1 | 0.68 (0.53, 0.88) | 0.57 (0.42, 0.77) | 0.60 (0.46, 0.78) | 0.74 (0.58, 0.95) | 0.01 |
| Cancer-attributed | 1 | 0.70 (0.55, 0.90) | 0.92 (0.72, 1.18) | 0.91 (0.74, 1.13) | 1.01 (0.82, 1.24) | 0.34 |
| Other-cause | 1 | 0.84 (0.66, 1.06) | 0.87 (0.67, 1.12) | 0.88 (0.71, 1.10) | 0.89 (0.71, 1.12) | 0.35 |
| Wine (n=375) |  |  |  |  |  |  |
| Alcohol consumption, g/wk | 0 | 1–25 | 26–150 | 151–350 | >350 | P for trend |
|  | n=58706 | n=305 | n=58 | n=10 | n=2 |  |
| CVD, cancer, and mortality | 1.00 (ref) | 0.88 (0.64, 1.22) | 1.26 (0.75, 2.13) | - | 5.06 (0.71, 35.96) | 0.26 |
| CVD | 1.00 (ref) | 0.87 (0.55, 1.39) | 1.25 (0.59, 2.63) | - | - | 0.58 |
| Myocardial infarction | 1.00 (ref) | 0.23 (0.03, 1.66) | - | - | - | 0.14 |
| Stroke | 1.00 (ref) | 0.76 (0.38, 1.52) | 1.06 (0.34, 3.30) | - | - | 0.55 |
| Heart failure | 1.00 (ref) | 1.32 (0.62, 2.79) | 1.95 (0.63, 6.09) | - | - | 0.77 |
| Atrial fibrillation | 1.00 (ref) | 1.20 (0.45, 3.25) | 2.17 (0.54, 8.81) | - | - | 0.77 |
| Cancer | 1.00 (ref) | 0.90 (0.47, 1.74) | 1.60 (0.60, 4.28) | - | 21.47 (3.00, 153.51) | 0.003 |
| Alcohol-related | 1.00 (ref) | 1.52 (0.62, 3.69) | 2.46 (0.61, 9.91) | - | - | 0.85 |
| Non-alcohol-related | 1.00 (ref) | 0.59 (0.22, 1.58) | 1.16 (0.29, 4.67) | - | 30.88 (4.31, 221.55) | <0.001 |
| Mortality | 1.00 (ref) | 0.70 (0.42, 1.17) | 0.98 (0.46, 2.05) | - | - | 0.40 |
| CVD-attributed | 1.00 (ref) | 0.58 (0.19, 1.80) | 1.27 (0.32, 5.10) | - | - | 0.80 |
| Cancer-attributed | 1.00 (ref) | - | 0.58 (0.08, 4.11) | - | - | 0.24 |
| Other-cause | 1.00 (ref) | 0.98 (0.41, 2.38) | 1.99 (0.64, 6.20) | - | - | 0.89 |

Values are hazard ratios and 95% confidence intervals.

Adjusted for age, sex, marriage (single, first marriage, other), education (elementary or below, middle or high school, college or above), household income per capita (≤500, 501–1000, >1000 Chinese yuan/month), smoking status (never, past, current), moderate/vigorous physical activity (<1, 1–3, ≥4 times/week), sodium intake (<6, 6–9.9, ≥10 g/day), body mass index (kg/m^2^), fasting blood glucose (mmol/L), anti-hypertension drugs (yes, no), anti-diabetes drugs (yes, no), and lipid-lowering drugs (yes, no).

CVD, cardiovascular diseases.

**Supplemental Table 10. Hazard ratios (95% confidence intervals) for CVD, cancer, and mortality by alcohol consumption tertiles.**

| Alcohol consumption, g/wk | 0 | 1–19 | 20–280 | >280 | P_trend_ |
| --- | --- | --- | --- | --- | --- |
|  | n=55446 | n=8151 | n=8583 | n=8292 |  |
| Composite CVD, cancer, and mortality | 1 (ref) | 0.70 (0.65, 0.76) | 0.85 (0.79, 0.91) | 0.95 (0.89, 1.01) | 0.008 |
| CVD | 1 | 0.73 (0.65, 0.81) | 0.83 (0.76, 0.91) | 0.95 (0.87, 1.05) | 0.02 |
| Myocardial infarction | 1 | 0.50 (0.38, 0.67) | 0.82 (0.67, 1.01) | 0.58 (0.46, 0.74) | <0.001 |
| Stroke | 1 | 0.78 (0.68, 0.90) | 0.86 (0.76, 0.97) | 1.15 (1.02, 1.29) | 0.26 |
| Heart failure | 1 | 0.66 (0.53, 0.84) | 0.69 (0.56, 0.85) | 0.60 (0.48, 0.75) | <0.001 |
| Atrial fibrillation | 1 | 0.97 (0.74, 1.26) | 1.00 (0.78, 1.27) | 1.15 (0.90, 1.47) | 0.40 |
| Cancer | 1 | 0.74 (0.63, 0.88) | 1.02 (0.89, 1.17) | 1.05 (0.92, 1.21) | 0.43 |
| Alcohol-related | 1 | 0.79 (0.60, 1.05) | 1.21 (0.96, 1.52) | 1.27 (1.01, 1.59) | 0.02 |
| Non-alcohol-related | 1 | 0.72 (0.59, 0.88) | 0.93 (0.79, 1.10) | 0.96 (0.81, 1.13) | 0.48 |
| Mortality | 1 | 0.63 (0.56, 0.71) | 0.79 (0.72, 0.87) | 0.85 (0.77, 0.93) | <0.001 |
| CVD-attributed | 1 | 0.62 (0.49, 0.78) | 0.59 (0.48, 0.74) | 0.71 (0.58, 0.88) | <0.001 |
| Cancer-attributed | 1 | 0.59 (0.47, 0.75) | 0.90 (0.75, 1.07) | 0.98 (0.82, 1.17) | 0.54 |
| Other-cause | 1 | 0.81 (0.66, 1.00) | 0.86 (0.71, 1.03) | 0.91 (0.75, 1.10) | 0.12 |

Values are hazard ratios and 95% confidence intervals.

Multivariate model adjusted for age, sex, marriage (single, first marriage, other), education (elementary or below, middle or high school, college or above), household income per capita (≤500, 501–1000, >1000 Chinese yuan/month), smoking status (never, past, current), moderate/vigorous physical activity (<1, 1–3, ≥4 times/week), sodium intake (<6, 6–9.9, ≥10 g/day), body mass index (kg/m^2^), fasting blood glucose (mmol/L), anti-hypertension drugs (yes, no), anti-diabetes drugs (yes, no), and lipid-lowering drugs (yes, no).

CVD, cardiovascular diseases.


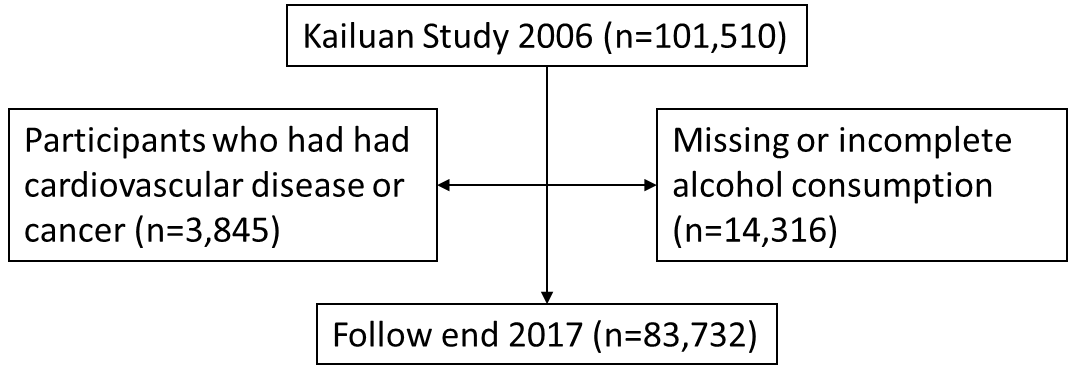


**Supplemental Figure 1. Flowchart of the study.**


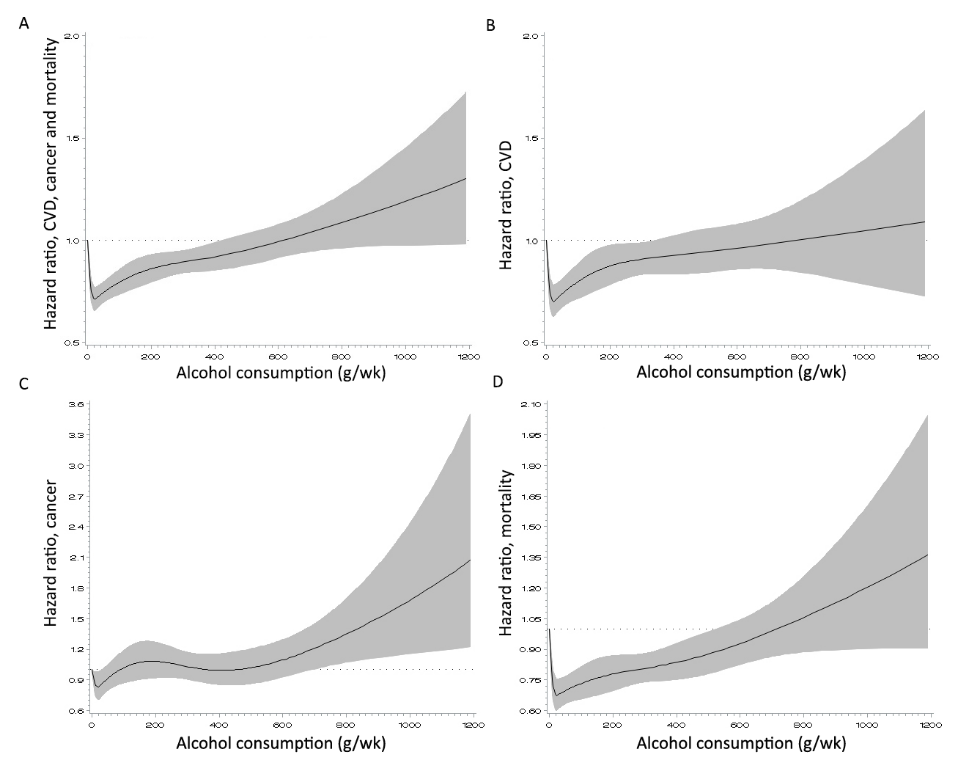


**Supplemental Figure 2. Restricted cubic spline model for alcohol consumption and composite CVD, cancer, and mortality (Panel A), CVD (Panel B), cancer (Panel C), and all-cause mortality (Panel D)**, adjusted for age, sex, marriage (single, first marriage, other), education (elementary school or below, middle or high school, college or above), household income per capita (≤500, 501–1000, >1000 Chinese yuan/month), smoking status (never, past, current), moderate/vigorous physical activity (<1, 1–3, ≥4 times/week), sodium intake (<6, 6–9.9, ≥10 g/day), body mass index (kg/m^2^), fasting blood glucose (mmol/L), anti-hypertension drugs (yes, no), anti-diabetes drugs (yes, no), and lipid-lowering drugs (yes, no). The reference category is zero consumption. Data plotted are hazard ratio. Cloud area represent 95% confidence intervals. CVD, cardiovascular diseases.
